# Editorial: Bioinformatics of Genome Regulation, Volume II

**DOI:** 10.3389/fgene.2021.795257

**Published:** 2021-11-08

**Authors:** Yuriy L. Orlov, Anastasia A. Anashkina, Tatiana V. Tatarinova, Ancha V. Baranova

**Affiliations:** ^1^ The Digital Health Institute, I.M.Sechenov First Moscow State Medical University (Sechenov University), Moscow, Russia; ^2^ Agrobiotechnology Department, Agrarian and Technological Institute, Peoples’ Friendship University of Russia (RUDN University), Moscow, Russia; ^3^ Engelhardt Institute of Molecular Biology, Russian Academy of Sciences, Moscow, Russia; ^4^ Natural Science Division, La Verne University, La Verne, CA, United States; ^5^ School of Systems Biology, George Mason University, Fairfax, VA, United States; ^6^ Research Centre for Medical Genetics, Moscow, Russia

**Keywords:** bioinformatics, transcriptomics, plant science, gene networks, gene expression regulation, genetics, computational genomics

This Research Topic Volume II, “Bioinformatics of Genome Regulation,” continues the studies in the field of bioinformatics of gene expression presented initially at *Frontiers in Genetics* journal (https://www.frontiersin.org/research-topics/8383/bioinformatics-of-genome-regulation-and-systems-biology) and then in Volume I (https://www.frontiersin.org/research-topics/14266/bioinformatics-of-genome-regulation-volume-i). The materials presented here were discussed in a BGRS\SB (Bioinformatics of Genome Regulation and Structure Systems Biology) conference series in Novosibirsk, Russia (https://bgrssb.icgbio.ru/2020/). The BGRS is the central event in the computational genetics field, held in Novosibirsk, Russia, every other year since 1998 (Orlov et al., 2015). The publications were later completed by new studies on computational methods of gene expression analysis regulation. Starting in 2018, materials of the conference materials in genetics and genomics were presented in *Frontiers in Genetics*, and due to popular demand in 2021 it was extended as the Volume II. The BGRS conference series have been presented at special journal issues earlier (Orlov et al., 2016; Tatarinova et al., 2019; Orlov et al., 2015; Orlov et al., 2019a; Orlov et al., 2019b; Baranova et al., 2019) and recently (Tatarinova et al., 2020; Orlov and Baranova, 2020; Orlov et al., 2020; Orlov et al., 2021a; Orlov et al., 2021b). We have to acknowledge “Bioinformatics of Gene Regulations and Structure” special issue at *MDPI IJMS* (https://www.mdpi.com/journal/ijms/special_issues/Bioinformatics_Genomics), as well as *PeerJ* journal BGRS-2020 collection (https://peerj.com/collections/72-bgrs-sb-2020).

This research topic presents seventeen papers on medical genomics applications, new bioinformatics tools and applications to laboratory animal models, and plant sciences.

Biomedical papers start from applications to cancer studies. Xiaorong Yang et al. discussed the interplay between human diseases at multiple biological levels. The authors show the role of schizophrenia in the pathological development of myocardial infarction, suggesting its role in promoting the development and progression of myocardial infarction at different levels, including genes, small molecules, and complex molecules. Pathway analysis revealed nine genes connecting these diseases.

Maxim Sorokin et al. proposed an algorithm that identifies functional roles of the pathway components and applied it to annotate 3,044 human molecular pathways extracted from the Biocarta, Reactome, KEGG, Qiagen Pathway Central, NCI, and HumanCYC databases. The resulting knowledgebase may be used to calculate the levels of activation for individual pathways and to establish large-scale profiles of the signaling, metabolic, and DNA repair regulation using high throughput gene expression data, as was presented recently (Wang et al., 2021).

Anna E. Letiagina et al. presented a new bioinformatics application on massively parallel reporter assays (MPRAs). The assays are based on the construction of reporter plasmid libraries. The authors present a pipeline for processing raw MPRA data obtained by NGS for reporter construct libraries with a priori unknown sequences of a region of interest and a barcode in the plasmid, located outside and within the transcription unit. The pipeline robustly identifies unambiguous barcodes, calculates the normalized expression level, and provides a graphical visualization of the processed data.

Maria M. Litvinova et al. have studied genes polymorphisms associations with calcium urolithiasis development. Authors found statistically significant associations between calcium urolithiasis and the polymorphisms in *CASR* rs1042636, *CALCR* rs1801197, and *ORAI1* rs6486795.

Yi He et al. explored the regulatory network among circRNA-miRNA-mRNA in osteosarcoma. The authors interrogated NCBI GEO osteosarcoma datasets and extracted circRNA, microRNA, and mRNA expression profiles. Downstream network analysis was performed with CircInteractome (Circular RNA Interactome) and mirDIP (microRNA Data Integration Portal) databases. Authors show that quinacridine, thalidomide, and zonisamide may be potential drugs for the treatment of osteosarcoma.

Stepan Nersisyan et al. investigated the effects of hypoxia on pathophysiological processes, including cancer progression and metastasis formation. This work continued the earlier studies on miRNA regulatory interactions in cancer (Shkurnikov et al., 2019). The landscape of hypoxia-induced miRNA and mRNA expression alterations was studied in human colorectal cancer cell lines (HT-29 and Caco-2) to show that miR-148a downregulation contributes to poor survival due to overexpression of the *ITGA5* and *PRNP* genes.

Sergey Nikulin et al.) have studied gene expression in breast cancer cells. The authors showed that malignant breast tumors with reduced expression of the *ELOVL5* and *IGFBP6* genes could metastasize at a higher probability due to a more efficient invasion of tumor cells. In addition, a set of novel computational techniques was developed for deciphering gene expression regulation.

Igor B. Rogozin et al. discussed fundamental biochemical mechanisms mutations in cancer driver genes. A large fraction of these mutations arose due to the off-target activity of DNA/RNA editing cytosine deaminases, followed by the replication/repair of edited sites by DNA polymerases. Using methylation data from malignant lymphomas, the authors showed that driver genes are subject to different (de)methylation processes than non-driver genes.

Natalya V. Klimova et al., using previously published web-tool SNP_TATA_Comparator (Ponomarenko et al., 2017), conducted a genome-wide study of single-nucleotide polymorphisms (SNPs) within core promoters of 68 human rheumatoid arthritis-related genes. They show that the disruptive natural selection of human immunostimulatory and immunosuppressive genes concurrently elevates and reduces the risk of rheumatoid arthritis. The authors hypothesize that rheumatoid arthritis in humans could be a self-domestication syndrome referring to evolution patterns in domestic animals (Chadaeva et al., 2021).

James Sweet-Jones et al. applied genetic tools to livestock breeding on the example of the Welsh mountain sheep breeds. Genotyping data from 317 individuals representing 15 Welsh sheep breeds were used alongside the whole-genome resequencing data of 14 species from the same set to scan for the signatures of selection and candidate genetic variants using haplotype SNP-based approaches. The authors found new variants in genes with potential functional consequences to the adaptation of local sheep to their environments in Wales. The study continues in new markers search in animal adaptation to cold climate (Igoshin et al., 2021).

Lilit Nersisyan et al. investigated telomere maintenance mechanisms for studying cancers and designing therapies.

In Brief Research Report, Elena V. Ignatieva et al. presented a new database - a catalog of human genes associated with pathologies of the sperm. It contains data genes related to male fertility and their functional annotation based on the literature data and clinical trials (Kolmykov et al., 2021). Functional annotation of genes from the catalog showed that spermatogenic failure could be associated with mutations in genes that control biological processes essential for spermiogenesis (such as DNA metabolism, cell division). Azoospermia can be caused by mutations in genes that control cellular responses to unfavorable conditions (stress factors, including oxidative stress and exposure to toxins).

Ekaterina Ilgisonis et al. considered a problem of transcriptome annotation by sequencing. They compared the results obtained from different transcriptome analysis platforms (quantitative polymerase chain reaction, Illumina RNASeq, and Oxford Nanopore Technologies MinION) for the transcriptome encoded by human chromosome 18 using the same sample types. The combination of Illumina RNASeq and MinION nanopore technologies reduced the probability of false-positive detection of low-copy transcripts due to the simultaneous confirmation of the presence of a transcript by the two fundamentally different technologies: short reads essential for reliable detection and long-read sequencing data.

The next group of articles in this Research Topic performed gene expression analysis in plants. This science field was presented at the bioinformatics conference series in Novosibirsk (Computer plant biology Session of BGRS conference, and the Plantgen conference series, https://conf.icgbio.ru/plantgen2021/) (Orlov, 2019; Orlov et al., 2019b).

Nann Miky Moh Moh et al. studied miRNAs and lncRNAs from the mango (*Mangifera indica* L.). Although mango is a popular food having pharmacological potential, its non-coding RNA data were limited. For the first time, a large-scale study identified nearly a hundred miRNAs and over 7,000 temperature-responsive lncRNAs. Characterization of target genes for these ncRNAs was performed.

Ekaterina M. Dvorianinova et al. presented the application of sequencing technologies to study plant pathogens of flax (*Linum usitatissimum* L.). Genome Assembly of the pathogen *Fusarium oxysporum* f. sp. *lini* was presented (Krasnov et al., 2020). Due to *F. oxysporum* f. sp. *lini* includes many genotypes, it is of high significance to study the origins of pathogenicity at the molecular level. This work mainly focused on genome sequencing of strains of the flax pathogen *F. oxysporum* f. sp. *lini*, possessing diverse pathogenicity degrees, on two platforms—Oxford Nanopore Technologies and Illumina. Sequencing using these two platforms proved to effectively achieve high-quality genome assemblies for complex plant genomes (Melnikova et al., 2021).

Victoria A. Scobeyeva et al. studied patterns of evolution in plant genome on the example of *Allium* species. *Alliums* are widespread and diversified; they are adapted to various habitats, from shady forests to open steppes. The genes present in chloroplast genomes (plastomes) play fundamental roles for photosynthetic plants. Plastome traits could thus be associated with geophysical abiotic characteristics of habitats. The authors compared their data with previously published plastomes and provided our interpretation of *Allium* plastome genes’ annotations. They can hypothesize that adaptive evolution in genes, coding subunits of NADH-plastoquinone oxidoreductase could be driven by abiotic factors of alpine habitats, especially by intensive light and UV radiation.

Elena N. Pushkova et al. studied Allele-Specific Expression in plants. Transcriptome sequencing of plant tissues from the male and female trees of *Populus* × *sibirica* and genome sequencing of the same plants were performed first. Targeted sequencing of sex-determining region (SDR) genes such as *CLC* (Chloride channel protein CLC-c on a representative set of trees confirmed the sex-associated allele-specific expression in generative and vegetative tissues of *P.* × *sibirica*.

Overall, we are proud of the continuing Research Topic at Frontiers in Genetics we collated. Biomedical applications for gene expression studies in chronic diseases are presented in ongoing Research Topic “High-throughput sequencing-based investigation of chronic disease markers and mechanisms” (https://www.frontiersin.org/research-topics/21036/high-throughput-sequencing-based-investigation-of-chronic-disease-markers-and-mechanisms). We hope you will find this paper collection a stimulating reading and consider coming to the next BGRS\SB conferences in Novosibirsk, Russia (https://bgrssb.icgbio.ru/2022/).
